# Repertoire of Computationally Designed Peroxygenases for Enantiodivergent C–H Oxyfunctionalization Reactions

**DOI:** 10.1021/jacs.2c11118

**Published:** 2023-01-23

**Authors:** Patricia Gomez de Santos, Ivan Mateljak, Manh Dat Hoang, Sarel J. Fleishman, Frank Hollmann, Miguel Alcalde

**Affiliations:** † Department of Biocatalysis, Institute of Catalysis, ICP-CSIC, C/ Marie Curie 2, 28049 Madrid, Spain; ‡ EvoEnzyme S.L., Parque Científico de Madrid, C/ Faraday 7, 28049 Madrid, Spain; § Chair of Biochemical Engineering, Technical University of Munich, Boltzmannstr. 15, 85748 Garching, Germany; ∥ Department of Biomolecular Sciences, Weizmann Institute of Science, 7610001 Rehovot, Israel; ⊥ Department of Biotechnology, Delft University of Technology, van der Massweg 9, 2629HZ Delft, The Netherlands

## Abstract

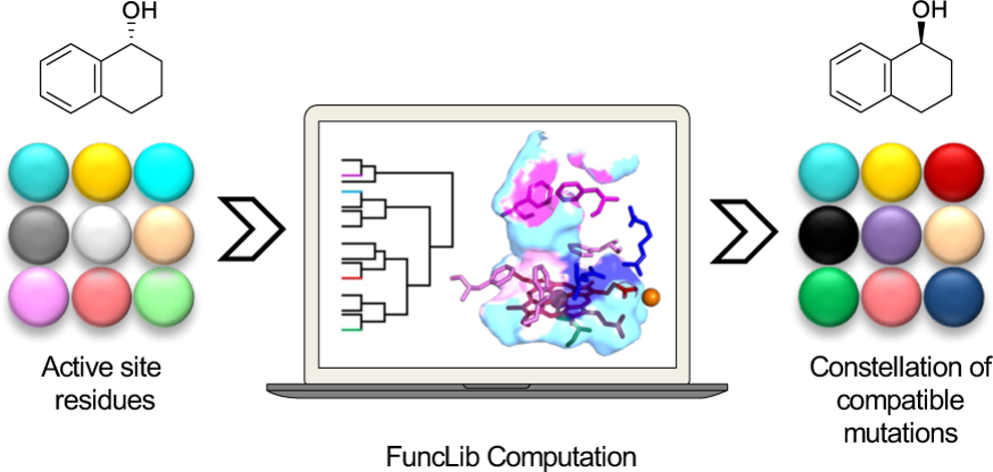

The generation of enantiodivergent biocatalysts for C–H oxyfunctionalizations is ever more important in modern synthetic chemistry. Here, we have applied the FuncLib algorithm based on phylogenetic and Rosetta calculations to design a diverse repertoire of active, stable, and enantiodivergent fungal peroxygenases. 24 designs, each carrying 4–5 mutations in the catalytic core, were expressed functionally in yeast and benchmarked against characteristic model compounds. Several designs were active and stable in a range of temperature and pH, displaying unprecedented enantiodivergence, changing regioselectivity from alkyl to aromatic hydroxylation, and increasing catalytic efficiencies up to 10-fold, with 15-fold improvements in total turnover numbers over the parental enzyme. We find that this dramatic functional divergence stems from beneficial epistasis among the mutations and an extensive reorganization of the heme channel. Our work demonstrates that FuncLib can rapidly design highly functional libraries enriched in enantioselective peroxygenases not seen in nature for a range of biotechnological applications.

## Introduction

Selective oxyfunctionalization reactions are considered by many as the “*holy grail*” of modern synthetic chemistry.^1,2^ Specially, the stereoselective oxyfunctionalization of non-activated C–H bonds remains a realm of biocatalysis, while improving the stereoselectivity in a given enzyme is principally achieved by protein engineering. However, inverting the enantioselectivity is a challenging task that requires complex rebuilding in the enzyme active site. Contemporary protein engineering approaches include *de novo* enzyme design, biohybrids, and directed evolution.^3−5^ The latter strategy is blossoming, thanks to novel genetic (*i.e.,* mutant library generation) and computational tools that seek to streamline resources and research effort. By mimicking the immense engineering power of natural evolution, directed evolution relies on iterative rounds of random mutation, DNA recombination, and artificial selection that are focused on a quite specific functional goal. Clearly successful when focusing on biochemical traits that can be coupled to high- or ultrahigh-throughput screening, the engineering potential of directed evolution is more limited when the mutant libraries have to be explored using low-throughput (analytical) methods—due to the lack of specific colorimetric/fluorimetric assays—as is the case when generating enantioselective C–H oxyfunctionalization biocatalysts.^6−8^ While the potential to use machine learning-guided evolution may lessen the experimental burden, these methods often demand preexisting experimental data on the specific protein system under study in order to generate useful predictions.^9^ By contrast, recent advances in computational engineering have allowed one-shot protein design based on a crystallographic structure and phylogenetic information.^10,11^ These methods are especially relevant to the design of enzymes that are difficult to characterize experimentally, such as the peroxygenases that are the focus of our current study. FuncLib is a powerful computational tool that combines Rosetta atomistic modeling and evolutionary conservation analysis to generate highly functional libraries of designs.^12^ The method uses evolutionary information to rule out mutations that are rarely observed in natural diversity and Rosetta atomistic calculations in order to eliminate destabilizing mutations. Finally, it harnesses Rosetta to nominate several dozen active-site designs that exhibit stable and preorganized constellations of amino acids within the catalytic core of the enzyme, Figure S1. Unlike structure-guided directed evolution, FuncLib does not target specific substrates and cannot do it since it does not model the substrate. The insight that underlies FuncLib is that the evolution of new activities is blocked by epistasis and that atomistic calculations may predict new constellations of multipoint active-site mutations compatible with protein folding and function (hence, potentially diverse and active). Since FuncLib produces many different constellations, it can unveil new latent/promiscuous activities in an unpredictable manner. FuncLib was originally conceived to exploit enzyme substrate promiscuity to derive variants that exhibit high activity toward substrates on which the parental enzyme exhibited only low activity (*i.e.,* to rescue latent/promiscuous activities). However, it has never been tested to create a catalytic portfolio of enantioselective mutants. In fact, enantioselectivity occurs in nature as an orthogonal biological function that demands a strong selective pressure and a stepwise specialization to be fully inverted. As FuncLib builds full reconfigured active-site networks, it is plausible to think that it could promote a catalytic enantio-switch, generating selectivities that do not exist in natural enzyme counterparts. Accordingly, we have used FuncLib to design an enantiodivergent biocatalytic repertoire for complex C–H oxyfunctionalization reactions. As a scaffold for this study, we chose the fungal unspecific peroxygenase (UPO, EC. 1.11.2.1) that is deemed to be the industrial replacement of long-studied cytochrome P450 monooxygenases (P450s) due to its ease of use, higher performance, and minimal requirements.^13−17^ The mutant parental UPO used in this work (referred to as PaDa-I) is a laboratory-evolved variant from *Agrocybe (Cyclocybe) aegerita* UPO—*Aae*UPO—,^18^ and it was subjected to FuncLib design in order to engineer functional multipoint active-site variants that were comprehensively benchmarked for enantioselectivity with a panel of representative compounds. Thus, 24 functional, highly stable, and diverse FuncLib designs were identified, exhibiting dramatic enantiodivergence and improvements up to 15-fold in total turnover numbers (TTNs) after just a single-shot computational design. Together, we showcase how FuncLib serves as a computational engineering platform for the rapid generation of multipoint active-site peroxygenase mutants with enantiodivergence for C–H oxyfunctionalization reactions.

## Results

### Design of a Diverse Peroxygenase Repertoire by FuncLib

**Figure 1:**
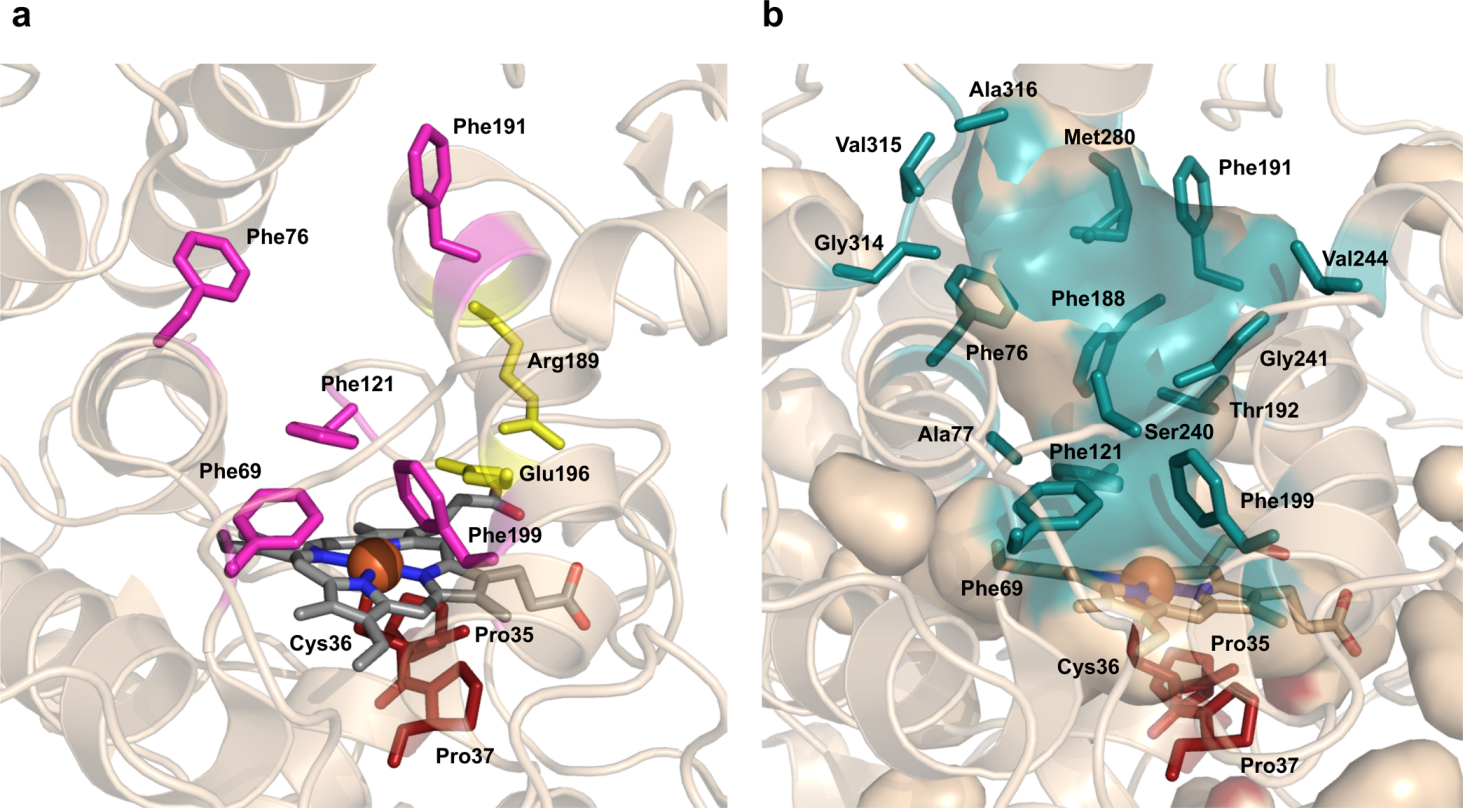
Overview of the PaDa-I crystal structure and selected residues for FuncLib mutagenesis. (a) Detail of the heme cavity with Phe76 and Phe191 at the entrance of the channel (in pink), the catalytic acid–base pair Glu196-Arg189 (in yellow), the Phe69–Phe121–Phe199 tripod (in pink), and the conserved Pro27–Cys36–Pro35 motif including the Cys axial ligand in red. The heme domain is depicted in gray with the iron cation of the catalytic intermediate compound I (^+•^Heme-Fe^4+^=O) as a sphere (PDB entry 6EKZ). (b) Surface and ribbon representations of 15 selected residues lining the heme channel (shadowed in teal) that have been subjected to FuncLib mutagenesis.

In order to obtain enantioselective mutants, directed evolution experiments are performed by structure-guided mutagenesis (mostly based on CAST—combinatorial active-site saturation test, ISM—iterative saturation mutagenesis, CAST/ISM, and more recently FRISM—focused rational iterative site-specific mutagenesis) in which the mutant libraries are screened with low-throughput analytical methods^19−21^ and references therein. By contrast, FuncLib is a computational algorithm that directly introduces multipoint compatible mutations into the enzyme pocket in a single shot, thereby saving considerable experimental effort.^12^ Given that inserting a large number of mutations into the active site may compromise enzyme stability, we attempted to stabilize the enzyme by applying the PROSS stability design algorithm prior to performing FuncLib mutagenesis.^22^ Using similar principles as FuncLib, PROSS focuses on the insertion of multiple stabilizing mutations at the protein surface rather than the catalytic site, with the aim of enhancing both stability and heterologous functional expression. We first ran PROSS on the PaDa-I mutant,^18^ selecting the most promising designs for their synthesis and cloning into *Saccharomyces cerevisiae*, Table S1. These variants were then characterized in terms of secretion, activity, and stability, both over a range of temperatures and in the presence of organic solvents, Figure S2a–d. None of the variant designs, which included from 5 to 14 mutations at the protein surface, considerably improved the stability or expression of the parental enzyme. However, it should be noted that the PaDa-I mutant is already a highly stable variant. Indeed, this enzyme is the product of five rounds of directed evolution for heterologous functional expression in *S. cerevisiae* and *Pichia pastoris*, and it carries a selected backbone of beneficial stabilizing mutations that improve expression, activity, and stability (*i.e.,*F[12]Y–A[14]V–R[15]G–A[21]D–V[57]A–V57A–L67F–V75I–I248V–F311L: the underlined residues lie in the leader sequence).^18^ Recently, these mutations have been adopted to stabilize and express other fungal peroxygenases from different sources.^23^ Since PROSS mutagenesis did not enhance the stability of PaDa-I, we assumed that our template was ready to tolerate an aggressive FuncLib campaign of mutagenesis and proceeded accordingly. On the basis of mutagenesis, soaking crystallography, computational quantum mechanics/molecular mechanics, and ligand diffusion studies, we previously found that the amino acids lining the PaDa-I heme access channel play a major role in the enzyme’s activity and selectivity.^24−26^ This heme channel is highly dynamic, with several possible conformations in regions that affect the mode of substrate binding. Such a malleable channel is mostly governed by aromatic amino acids, with two apical Phe residues (Phe76 and Phe191) delimiting the entrance to the heme cavity and a tripod formed by Phe69, Phe121, and Phe199 that is crucial to situate the substrate at a van der Waals distance from compound I, the ferryl-oxo complex, and the reactive key intermediate in the C–H insertion of oxygen, Figure 1a.

We applied FuncLib to 15 selected amino acids that make up the main part of the channel, Figure 1b, Table S2. During all FuncLib calculations, we held fixed the conformations of amino acid residues in charge of attaching the heme prosthetic group, the critical catalytic acid–base pair (Glu196–Arg189) involved in the heterolytic cleavage of H_2_O_2_, and the amino acids responsible for the binding of the characteristic structural magnesium cation. Based on its phylogenetic analysis and Rosetta atomistic design calculations, FuncLib automatically ranked mutants harboring combinations of 4–5 active-site mutations. From the top 50 designs, we selected a sample of the top 22 mutants with the lowest energy and eight handpicked designs, Table 1. The latter were selected to assess the potential beneficial epistasis of the F121Y, F199L/A, and F76Y mutations within the different constellations of mutations returned by the algorithm. The prevalence of the F199L mutation and the A316P mutation was noteworthy. Interestingly, when Ala316 was previously subjected to saturation mutagenesis, the A316P mutant was seen to be associated with an improvement in the *k*_cat_/*K*_m_ in the range of 1.5- to 6-fold for all the substrates tested.^25^ By contrast, up to four positions were maintained unaltered in all the variants (Phe188, Phe191, Met280, and Val315), which is notable, as in directed evolution studies Phe191 and Val315 were seen to influence regioselectivity and activity, respectively.^24,27^

**Table 1:**
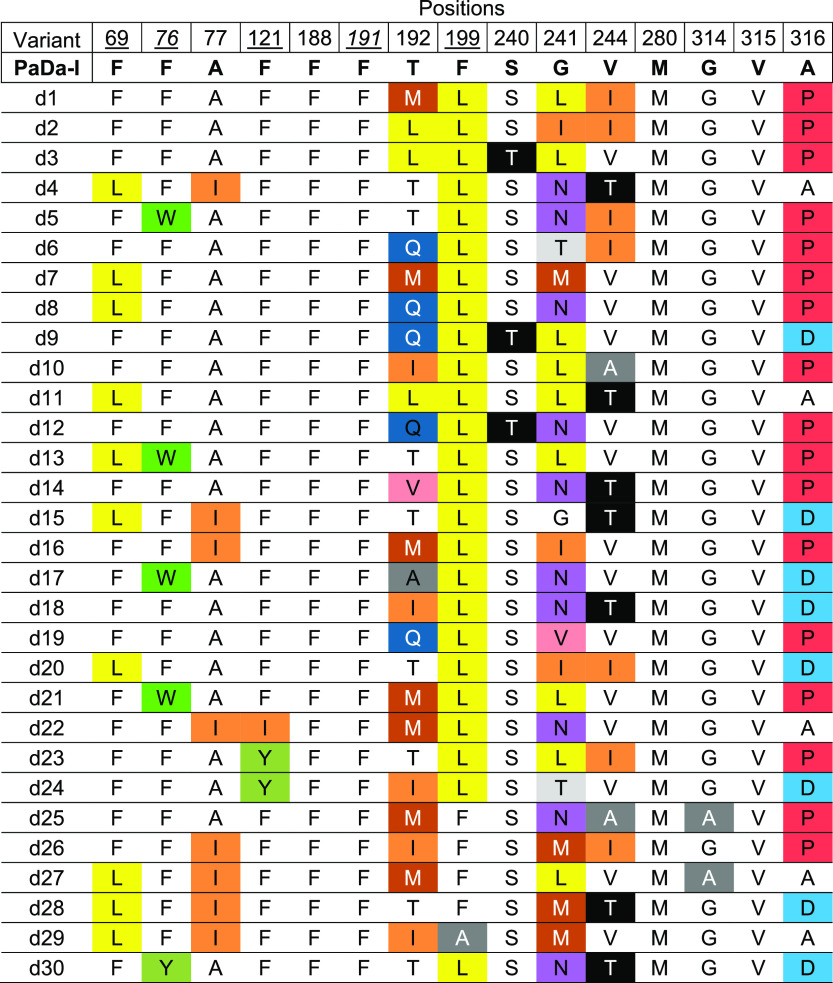
FuncLib UPO Designs^a^

a Targeted positions and their corresponding amino acid substitutions in each FuncLib design. Underlined the Phe tripod in charge of positioning the substrate and underlined in italics the two apical Phe at the entrance of the heme channel. Mutations are highlighted in different colors.

The 30 FuncLib designs were synthesized, cloned *in vivo* with the help of overlapping flanking regions, and expressed in *S. cerevisiae*. Activity was first evaluated with common colorimetric substrates: ABTS (2,2′-azino-bis(3-ethylbenzothiazoline-6-sulfonic acid)) and DMP (2,6-dimethoxyphenol) for peroxidase activity (one electron oxidation reaction); and NBD (5-nitro-1,3-benzodioxole) for peroxygenase activity (a peroxide-borne oxygen-transferring, two-electron oxidation reaction), Figure S3. Remarkably, of the 30 FuncLib designs, 24 were functionally expressed, while only d6, d16, d22, d23, d24, and d27 were not functional. This result represents a very high ratio of success in generating functional multipoint active-site variants in comparison to conventional methods like rational design or combinatorial saturation mutagenesis.

Most of the variants displayed weaker activity on ABTS, with the exception of d25 and d28; by contrast, the peroxygenase activity against NBD was the highest for 18 designs, a substrate that must be placed within a van der Waals distance from the deeply buried catalytic intermediate compound I. Compared to PaDa-I, the peroxygenase/peroxidase activity ratio was notably altered, indicating a broad potential for functional diversity within the catalytic repertoire of FuncLib. Functional expression was reduced to a greater or lesser extent depending on the mutant and the activity evaluated, a property that was fully recovered by transferring the designs from *S. cerevisiae* to *P. pastoris* (*vide infra*).

### Strong Enantiodivergence Among FuncLib Designs

**Figure 2:**
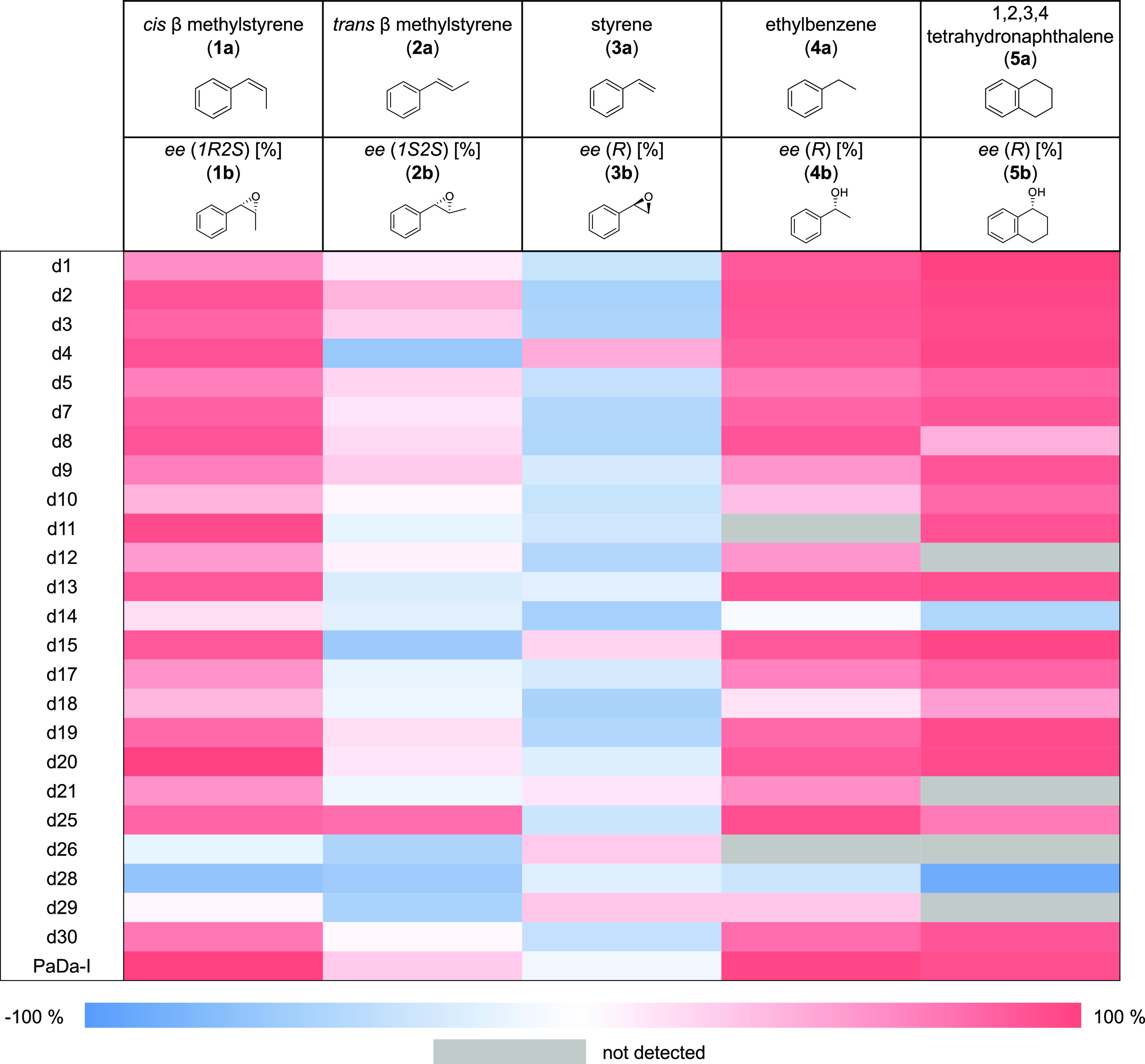
Heat map of ee of FuncLib designs compared to parental-type PaDa-I. See also Tables S3, S4 and Figure S4.

Engineering enantioselective enzymes for C–H oxyfunctionalization reactions usually requires iterative cycles of directed evolution limited by low-throughput screening and time-consuming chiral chromatographic analysis methods, allowing only a very minor fraction of the mutant library to be screened.^2,6^ Here, we constructed by FuncLib computational mutagenesis a high-quality library of functional designs, whose divergence was analyzed with a panel of representative model compounds. Our main purpose was to determine if such a catalytic repertoire, generated after a single-shot computational multipoint design process, could provide versions of the same enzyme with altered enantioselectivity. Pleasingly, several designs inverted enantioselectivity to a greater or lesser extent for each of the substrates tested: *cis* β methylstyrene (**1a**), *trans* β methylstyrene (**2a**), styrene (**3a**), ethylbenzene (**4a**), and 1,2,3,4-tetrahydronaphthalene (**5a**), Figures 2, S4, Tables S3, S4.

The parental PaDa-I exhibits high enantiomeric excess (ee > 90) for substrates **1a**, **4a,** and **5a**, whereas it produces almost racemic product mixtures with **2a** and **3a**. Thus, while PaDa-I transforms **1a** into **1b** (1*R*2*S*) with an ee > 99%, several designs clearly tended to invert enantioselectivity, with the d28 variant producing 83% of **1c** (1*S*2*R*). PaDa-I also has excellent enantioselectivity, converting **4a** and **5a** into the corresponding *R* enantiomers with ee values of 98 and 94%, respectively. With these two substrates, the d28 mutant showed a noticeable switch in enantioselectivity, generating the corresponding (*S*)-enantiomers (**4c**-*S*-phenylethanol and **5c**-(*S*)-(+)-α-tetralol) in 32 and 86% ee, respectively. Also, other designs exhibited a notable inversion of enantioselectivity (d8, d14, d18). Besides this switch in enantioselectivity, some designs also changed their regioselectivity (*e.g.,* from side-chain hydroxylation to aromatic hydroxylation), a reaction never observed in wildtype *Aae*UPO with **4a**, whereby d7, d8, and d13 produced *o*-hydroxy-ethylbenzene, and d1, d2, d3, d4, d5, d10, d14, d18, and d30 produced *p*-hydroxy-ethylbenzene to a greater or lesser extent, Tables S3, S4. Similarly, many of the designs changed the regioselectivity for **5a** to position 2, Tables S3, S4. Given that **2a** and **3a** are transformed by PaDa-I with poor enantioselectivity, yielding almost racemic mixtures (*i.e.,* 66% of **2b** (1*S*2*S*) and 47% of **3b** (*R*), respectively (Table S4)), we expected to find several versions of the enzyme with divergent enantioselectivities for these two substrates. Indeed, with **2a,** we identified mutants producing >70% of **2c** (1*R*2*R*) (d4, d15, d26, d28, and d29) or **2b**—1*S*2*S*—(d2 and d25), Figure S2 and Table S4. Likewise, with **3a,** several designs produced over 70% of **3c** (*S*) (d2, d3, d7, d8, d12, d14, d18, and d19) or **3b** (*R*) (d4). As a clear signal of the broad versatility of this catalytic repertoire, two of the FuncLib designs had dramatic opposed enantioselectivity for both compounds, with the d2 mutant producing **2b** in 44% ee and **3c** in 52% ee, whereas d4 produced **2b** in 62% ee and **3b** in 48% ee, Figure 2, Table S3.

### Designs Exhibit High Activity, Stability, and Expressibility in *P. pastoris*

The parental PaDa-I and the FuncLib variants d2, d4, and d28 were produced, purified, and characterized biochemically. Given the reduced UPO expression observed in *S. cerevisiae* after FuncLib mutagenesis, these mutants were transferred to *P. pastoris* (*Komagataella phaffii*), which is a well-known host for peroxygenase production under the control of strong promoters.^28,29^ We applied a strain screening protocol based on increasing amounts of the antibiotic zeocin, such that optimal multicopy variants were isolated and used to carry out fermentations in a 5 L fed-batch bioreactor. With this system, UPO production increased up to ∼0.2 g/L, a level of expression similar to that of the parental PaDa-I in this host.^29^ PaDa-I, d2, d4, and d28 from *P. pastoris* were purified to homogeneity, and their thermostability, optimum pH activity, pH stability, as well as their kinetic and spectroscopic properties, were assessed, as shown in Table 2 and Figures S5–S7. Although the variants had similar biochemical properties, some differences in kinetic thermostability were observed (*e.g.,* d28 had a *T*_50_ value of 3 °C above that of the parental PaDa-I), as well as in their activity and stability to pH (*e.g.,* d4 had a broader pH activity profile from 4.0 to 9.0, whereas d28 shifted its optimum pH value for NBD from 6.0 to 7.0). Significantly, d4 was the most stable mutant in the pH range of 3.0 to 9.0, retaining its activity after a 22 h incubation. We estimated the kinetic parameters for ethylbenzene, the only model compound studied that releases a measurable product upon oxidation by UPO without any overlap with the substrate in the UV/vis range, which allowed us to develop a reliable kinetic spectrophotometric assay, Figure S8, Table 2.

**Table 2:** Biochemical and Spectroscopy Features of Purified PaDa-I and FuncLib Designs Expressed in *P. pastoris*^a^

feature	PaDa-I	d2	d4	d28
thermal stability, *T*_50_ (°C)	55.2	54.0	54.6	58.1
optimum pH for ABTS	4.0	4.0	4.0	4.0
optimum pH for DMP	5.0–6.0	6.0	8.0	6.0
optimum pH for NBD	6.0	6.0	5.0	7.0
*R*_Z_, (*A*_418_/*A*_280_)	1.2	1.9	1.4	1.8
Soret region (nm)	417	419	423	419
CT1 (nm)	570	568	568	568
CT2 (nm)	537	537	538	538
*K*_m_ (mM)*	16 ± 6	0.56 ± 0.2	0.56 ± 0.2	2.02 ± 0.7
*k*_cat_ (s^–1^)*	3914 ± 1043	570 ± 119	1412 ± 229	884 ± 156
*k*_cat_/*K*_m_ (mM^–1^s^–1^)*	244	1019	2521	438

a Rz, reinheitszahl value; CT1 and CT2, charge transference bands 1 and 2, respectively. *Apparent kinetic constants for ethylbenzene were determined using a calculated extinction coefficient for phenyl ethanol (ε_248_) = 147.3 M^–1^ cm^–1^ (Figure S8). See also Figures S5–S7.

Both d2 and d4 designs, with similar enantioselectivity as that of PaDa-I for ethylbenzene, showed a remarkable 28-fold increase in affinity (*K*_m_) toward this compound. Although this was not accompanied by an improved *k*_cat_, catalytic efficiencies were up to 10-fold higher than those of PaDa-I. By contrast, d28 switched enantioselectivity and increased affinity roughly eightfold, displaying a twofold higher *k*_cat_/*K*_m_ than the parental PaDa-I. In addition, we measured the TTNs after a 3 h reaction in the presence of 2 mM H_2_O_2_ with **5a** for d28 and with **3a** for d2 and d4. PaDa-I produced a TTN (expressed as μmol product/μmol enzyme) of 2300 for **5b**, while d28 yielded a TTN of 13,800 for **5c**, constituting a sixfold improvement in an enantiodivergent reaction. In the case of **3a**, both PaDa-I and d2 had a TTN of ∼450, with d4 reaching a TTN of 7100, a striking 15-fold improvement in the production of the *R* enantiomer after one single experiment of multipoint mutagenesis.

### Structural Basis of Enantiodivergence in FuncLib Designs

**Figure 3:**
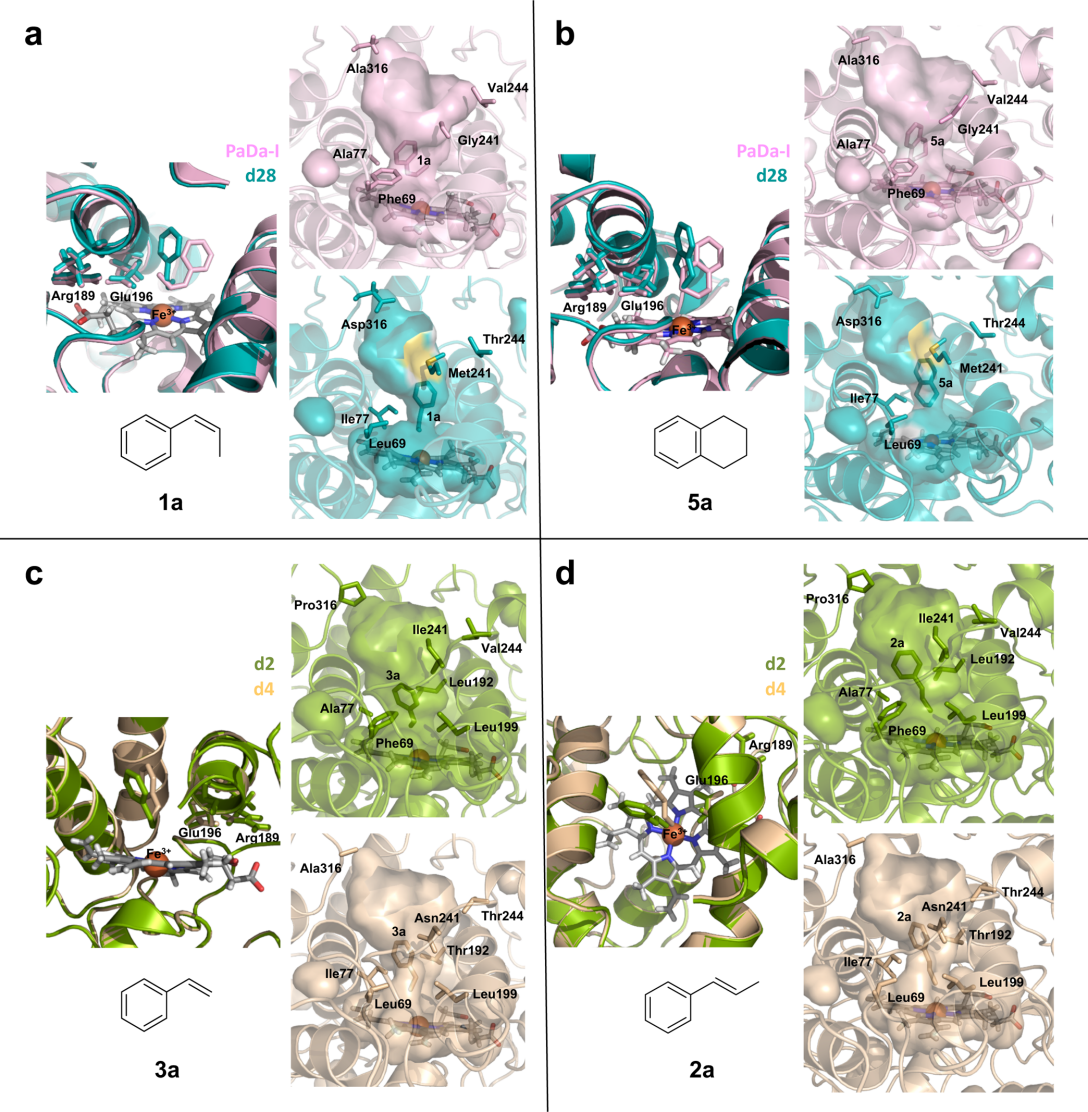
Comparison of docking studies with different substrates (**1a**, **5a**, **3a**, **2a**) for PaDa-I (pink), d28 (teal), d2 (green), and d4 (wheat) designs.

The different degrees of enantioselectivity observed among the mutants are a direct consequence of the composition and synergies established between the mutations included in each design. We tried to rationalize these dramatic changes in enantioselectivity through molecular docking simulations, Figure 3. Starting with d28 (carrying FuncLib mutations F69L–A77I–G241M–V244T–A316D) and its reaction with **1a**, the positioning of this substrate within the heme is limited due to the narrowing of the access channel imposed by mutation A77I. However, this substitution alone cannot explain the strong shift in selectivity, as A77I was also present in other designs (d4 and d15) that conserved the enantioselectivity of the parental PaDa-I, as shown in Table 1, Figure 2. Therefore, rather than a single mutational effect, synergy among a combination of mutations was noted, with the strongest modifications coming from F69L and G241M, which together with the aforementioned A77I notably contribute to reshaping the malleable active site of UPO, allowing the substrate to be placed in a distinct orientation relative to the parental PaDa-I, Figure 3a. Such reconfiguration of the channel is also responsible for the strong enantiodivergence of d28 with other model compounds, such as with **5a** that gives rise to 93% of **5c** as opposed to 97% of **5b** produced by PaDa-I. According to our simulations, the positioning of **5a** in d28 is restricted by the mutations, shifting the orientation toward the heme and the acid–base pair involved in the heterolytic cleavage of H_2_O_2_, Figure 3b. The considerable plasticity of the UPO heme channel was also observed after modeling the binding of the structurally related **2a** and **3a**. For these two compounds, d2 and d4 designs (carrying the mutations T192L–F199L–G241I–V244I–A316P and F69L–A77I–F199L–G241N–V244T, respectively) were seen to have opposed enantioselectivity, Figure 2. The positioning of **2a** and **3a** in both design models reflected a 45° twist relative to each other, Figure 3c,d. This twist is modulated by the shape of the heme access channel, mainly driven by the residues at positions 69, 77, 192, 199, and 241. Our simulations indicated that these amino acids are of paramount significance in the substrate binding mode. Phe69 and Phe199 are part of the aromatic tripod responsible for positioning the substrate at a catalytic distance from the heme, along with Phe121, the latter remaining virtually unaltered in the sequence space calculation of the FuncLib algorithm. As such, a reconfiguration of the heme channel occurs when the two former Phe residues are mutated together with Ala77, which helps narrow the substrate entrance to the heme cavity to some degree, as we have recently reported for the selective hydroxylation of fatty acids and alkanes.^30^ Alternatively, Gly241, Val244, and Ala316 are parts of highly dynamic loops (the Ser240–Asp245 and Gly314–Gly318 loops, respectively) that may exert a strong influence on substrate trafficking to the heme. Indeed, we modified these residues in previous directed evolution campaigns, altering the activity of the variants as a result of adopting different conformational states.^24,26,27,31^ Finally, the interaction with the alkyl double bond of a model compound also seems to be crucial, as this switch in selectivity is not seen with **4a**, even considering the structural similarity between these substrates.

## Discussion

Stepwise accumulation and recombination of random mutations underlie natural and directed evolution. While a highly efficient engineering process, natural molecular evolution is slow. To be able to glean the benefits of natural selection in the laboratory, directed evolution compresses the time frame of evolution and enables what would otherwise take millions of years to be performed over a manageable experimental period. Despite the benefits in terms of protein engineering, directed evolution is limited in tackling the design of enantioselective C–H oxyfunctionalization biocatalysts due to the obligation to use low-throughput (analytical) screening. In addition to this bottleneck, the construction and exploration of combinatorial saturation mutagenesis libraries far exceed the capacities of laboratories, leading us into a scenario in which the useful sequence space is barely explored for any given activity. Ultimately, in directed evolution, the protein alphabet is applied to mutagenesis, and the number of possible substitutions and recombination events is dramatically constrained due to the high epistasis within an enzyme active site.

Because of their significance in synthetic chemistry, UPOs and, in particular, the *Aae*UPO have been subjected to directed evolution for more than 10 years now, although they have never been engineered for enantioselectivity.^28^ Here, after one application of FuncLib design, 24 active and stable designs have been generated, with up to five substitutions in the catalytic core and a striking enantiodivergence difficult to achieve by conventional directed evolution as it depends on reciprocal sign epistatic mutations, which are exceedingly complex for an evolutionary process to unveil.

It is important to highlight that FuncLib works without transition-state modeling, that is, it does not perform ligand simulations at the active site but rather implements stable constellations of interacting amino acids in the catalytic core. The only requirement is a crystallographic structure of the enzyme. Interestingly, we have recently demonstrated that deep-learning-based ab initio structure prediction methods can overcome the requirement for crystallographic structures when applying PROSS stability design.^32^ The high accuracy observed in the active-site pockets of AlphaFold models relative to crystallographic structures suggests that FuncLib design could also be applied to such models. Thus, optimizing the stability, expressibility, and activity profiles of enzymes can free them from many of the most significant bottlenecks that have limited protein engineering for decades.

Despite its large hydrophobic content, the frustum cone-shaped channel of the UPO used in our study is highly malleable and flexible, offering a great potential to engineer new functions. In this regard, it is worth noting that d4 and d28 mutants were the most stable variants in response to changes in pH and temperature, and despite presenting divergent enantioselectivities for most of the model compounds, they both had the highest TTNs with a narrower heme channel compared to parental-type PaDa-I or d2. Thus, these FuncLib designs do not exhibit the trade offs between stability and activity that are often observed in other protein engineering campaigns. This is likely due to the fact that FuncLib selects low-energy designs for experimental testing to generate useful diversity. While their improved stability may help achieve higher TTNs, this characteristic conformation of the heme channel allowed the substrate to be placed in specific poses that promote enantioselective oxygen transfer from compound I, as seen in our docking studies. Both d4 and d28 share three mutations (F69L, A77I, and V244T) that can contribute to enhanced activity beyond what PaDa-I or d2 have. Even with these shared mutations, the other two specific mutations (F199L/G241N in d4 and G241M/A316P in d28) contribute to the big differences in selectivity between the mutants. All in all, d28 represents a beautiful example of an enantiodivergent peroxygenase, as this design possesses enantiocomplementary activity with 4 of the 5 substrates tested when compared to PaDa-I, as well as higher levels of activity and stability, which make a suitable point of departure for further laboratory evolution studies.

Finally, it is hard to determine the effect of each individual mutation as the substitutions introduced in the FuncLib designs have complex epistatic relationships, such that the effects of each one cannot be predicted in terms of single-point mutants.^12,33^ In this regard, we previously studied the three Phe residues at the bottom of the heme channel (in charge of orienting the substrate for catalysis) by saturation (combinatorial) mutagenesis, and the emerging mutant library was mostly inactive (∼80% of the clones).^24^ By contrast, the FuncLib library included 24 functional designs with inverted enantioselectivity and/or improved activity and stability in one single round of multipoint mutagenesis that, in many cases, included mutations of these Phe residues in combination with other substitutions along the heme channel. When analyzing the sequence space computed by the FuncLib algorithm, only five possible mutations were considered (F69L, F121I/W/Y, and F199A/L/Y), and the possibilities became even more limited after the final scoring by energy (F69L, F121I/Y, and F199A/L). From the activity results, we conclude that Phe121 is a residue with low tolerance to mutation since all variants mutated at this position were inactive. By contrast, the F69L mutation was present in the two most active variants, and the F199L mutation was the most common change suggested by the algorithm and present in 19 of the 24 active FuncLib mutants. As such, the Phe tripod seems to tolerate a number of substitutions in conjunction with other mutations, with clear preferences for aliphatic amino acids; these alter the conformation of the channel while maintaining the characteristic hydrophobic environment of this catalytic site.^25^

## Conclusions

FuncLib was originally conceived to generate catalytic repertoires of enzymes with latent/promiscuous activities, and in a very short time, it has been successfully applied to very different situations, ranging from the design of regioselective pyrazole alkylating methyltransferases^33^ to the engineering of cytoplasmic-soluble N-glycosyltransferases with narrow substrate specificity,^34^ the enhancement of the catalytic efficiency in a *de novo* Kemp eliminase,^35^ the increase in the reactivity profile of high-redox potential laccases,^36^ or to the stabilization of the SARS-CoV-2 spike receptor binding domain.^37^ We view FuncLib as a method for computational mutagenesis that can help address complex enzyme engineering problems, and here, we demonstrate that FuncLib represents an efficient and rapid approach to generate small but highly functional libraries enriched in enantioselective peroxygenase mutants for complex C–H oxyfunctionalization reactions, which, in the future, could be assisted by machine learning-guided evolution to search for optimum beneficial epistasis among mutations.^7,9^ The opportunity of having a *palette* of UPO variants with different enantioselectivities will pave the way for the generation of novel compounds and catalytic pathways to be used in applications ranging from lead diversification to the synthesis of fine chemicals.

## Materials and Methods

### Strains and Chemicals

Zeocin was purchased from Invitrogen (USA). The *Escherichia coli* strain XL2-Blue competent cells were obtained from Agilent Technologies (USA). The uracil-independent and ampicillin-resistant shuttle vector pJRoC30 was from the California Institute of Technology (CALTECH, USA). The protease-deficient *S. cerevisiae* strain BJ5465 was obtained from the LGCPromochem (Barcelona, Spain). The *P. pastoris* strain BG11 was purchased to Atum (USA). The plasmid used (pBSY5Z) was provided by Bisy (Austria). Restriction endonucleases *Eco*RI, *Xba*I, *Pme*I, *Bam*HI, and *Xho*I; the DNA Ligation Kit; the Antarctic phosphatase; and the PNGase F were purchased from New England Biolabs (USA). iProof High-Fidelity DNA Polymerase was purchased from Bio-Rad (USA). Oligonucleotide primers and UPO genes were acquired from Integrated DNA Technologies (USA). The NucleoSpin plasmid kit and NucleoSpin Gel and PCR Clean-up kit were purchased from Macherey Nagel (Germany). ABTS was purchased to Panreac AppliChem (Germany), and DMP and NBD were purchased to TCI Europe (Switzerland). H_2_O_2_, styrene, *trans* β methyl styrene, *cis* β methyl styrene, 1,2,3,4-tetrahydronaphtalene, and ethylbenzene were purchased from Merck Life Science (USA). All chemicals and medium components were of the highest purity available.

### Computational Design

PROSS mutants were designed with default settings (https://pross.weizmann.ac.il/step/pross-terms/)^22^ using the PaDa-I crystal structure (PDB entry 6EKZ)^25^ as an input. Residues F191, F76, S240, G241, V244, G314, V315, A316, A57, F67, I75, V248, and L311 were fixed during calculations, as were magnesium and heme ligands.

FuncLib mutagenesis was performed as reported elsewhere (https://funclib.weizmann.ac.il/).^12^ On the base of the PaDa-I crystal structure (PDB entry 6EKZ)^25^ 15 amino acids comprising the heme channel were targeted for FuncLib mutagenesis (F69, F76, A77, F121, F188, F191, T192, F199, S240, G241, V244, M280, G314, V315, and A316). Both structural magnesium and heme ligands were maintained unaltered in the calculations. The multiple sequence alignment was done according to the default parameters, and the top 22 designs based on the energy score were selected together with eight more from the pool for further experimental analysis.

Molecular docking simulations were performed using the Autodock VINA^38^ algorithm included in YASARA-Structure software^39^ using the crystal structure of PaDa-I at a resolution of 1.08 Å (PDB entry 6EKZ). Docking computations were performed at the level of the YASARA force field by running a number of 100 docking trials. Models were visualized with the PyMOL Molecular Graphics System, Version 2.0 Schrödinger, LLC.

### Cloning of FuncLib Mutants in *S. cerevisiae*

The DNA sequences containing both PROSS and FuncLib designs with the evolved signal peptide from the PaDa-I mutant^18^ were synthetized (with overhangs to promote homologous recombination) and cloned under the control of the GAL1 promoter of the pJRoC30 expression shuttle vector using *Bam*HI and *Xho*I to linearize the plasmid and remove the parent gene. The linearized vector was loaded onto a preparative agarose gel and purified with the NucleoSpin Gel and PCR Clean-up kits. The corresponding gene (200 ng each) was mixed with the linearized plasmid (100 ng) and transformed into *S. cerevisiae* for *in vivo* gene reassembly and cloning. The plasmids were recovered with the Zymoprep yeast plasmid miniprep kit I. Since the products of the Zymopreps were impure and the DNA extracted was very poorly concentrated, the shuttle vectors were transformed into supercompetent *E. coli* XL2-Blue cells and plated onto Luria–Bertani (LB)–ampicillin plates. Single colonies were grown in 5 mL of LB–ampicillin medium and incubated overnight at 37 °C and 225 rpm. The plasmids were extracted (NucleoSpin plasmid kit), sent for DNA sequencing (GATC Biotech-Eurofins, Luxembourg), and transformed into *S. cerevisiae* for flask production.

### Production of FuncLib Mutants in *S. cerevisiae*

#### Culture Media

Sterile minimal medium for flasks contained 100 mL of 19.2 g/L filtered yeast synthetic drop-out medium supplement without uracil, 100 mL of 6.7% filtered yeast nitrogen base, 25 mL of filtered 20% glucose, 775 mL of ddH_2_O, and 1 mL of 25 g/L filtered chloramphenicol. SC drop-out plates contained 100 mL of 19.2 g/L filtered yeast synthetic drop-out medium supplement without uracil, 100 mL of 6.7% filtered yeast nitrogen base, 20 g autoclaved bacto-agar, 100 mL of 20% filtered glucose, 1 mL of 25 g/L filtered chloramphenicol and ddH_2_O to 1000 mL. Sterile expression medium contained 720 mL autoclaved YP, 111 mL 20% filtered galactose, 67 mL of 1 M filtered KH_2_PO_4_ pH 6.0 buffer, 31.6 mL of absolute ethanol, 22 mL of filtered MgSO_4_ 0.1 M, 1 mL of 25 g/L filtered chloramphenicol, and ddH_2_O to 1000 mL. YP medium contained 10 g yeast extract, 20 g peptone, and ddH_2_O to 650 mL. YPD solution contained 10 g yeast extract, 20 g peptone, 100 mL of 20% sterile glucose, 1 mL of 25 g/L chloramphenicol, and ddH_2_O to 1000 mL. LB medium was prepared with 5 g yeast extract, 10 g peptone, 10 g NaCl, 100 mg ampicillin, and ddH_2_O to 1000 mL.

#### Small-Scale Flask Fermentation

A single colony from each *S. cerevisiae* clone containing the FuncLib mutant was picked from a SC drop-out plate, inoculated in minimal medium for flasks (10 mL), and incubated for 48 h at 30 °C and 230 rpm. An aliquot of cells was used to inoculate minimal medium (10 mL) in a 100 mL flask (OD_600_ = 0.25). The cells completed two growth phases (6–8 h), and then the expression medium (9 mL) was inoculated with the pre-culture (1 mL) (OD_600_ of 0.1). After incubating for 72 h at 25 °C and 230 rpm (maximal UPO activity; OD_600_ = 25–30), the cells were recovered by centrifugation at 5000 rpm for 20 min (at 4 °C), and the supernatant was double-filtered (using both a glass membrane and a nitrocellulose membrane of 0.45 μm pore size) and concentrated.

### Colorimetric Screening Assays

ABTS activity was measured in 100 mM sodium phosphate/citrate buffer pH 4.0 containing 1 mM ABTS; DMP activity was measured in 100 mM phosphate buffer pH 6.0 containing 1 mM DMP; and NBD activity was estimated in 100 mM phosphate buffer pH 7.0 containing 1 mM NBD in acetonitrile 15% (v/v). Reactions were performed in triplicate, and substrate oxidations were followed through spectrophotometric changes (ε_418_ ABTS^•+^ = 36,000 M^–1^ cm^–1^; ε_469_ cerulignone = 27,500 M^–1^ cm^–1^ and ε_425_ 4-nitrocatechol = 9700 M^–1^ cm^–1^).

### Cloning and Production of FuncLib Variants in *P. pastoris*

The DNA sequence of clones d2, d4, and d28 with the evolved signal peptide from the PaDa-I mutant was cloned into the vector pBSY5Z and produced using the carbon source-repressed promoter P_*DF*_ as described before.^40,41^

### Purification of PaDa-I, d2, d4, and d28 Designs from *P. pastoris*

Recombinant UPO purification was achieved by cationic exchange chromatography and anion exchange chromatography (ÄKTA purifier, GE Healthcare, WI, US). The crude extract was concentrated and dialyzed in sodium phosphate/citrate 20 mM at pH 3.3 (buffer A) by tangential ultrafiltration (Pellicon; Millipore, Temecula, CA, US) through a 10 kDa-pore-size membrane (Millipore) by means of a peristaltic pump (Masterflex Easy Load; Cole-Parmer, Vernon Hills, IL). The sample was filtered and loaded onto a strong cation-exchange column (HiTrap SPFF GE Healthcare) pre-equilibrated with buffer A. The proteins were eluted with a linear gradient from 0 to 40% (in 40 min) of buffer A with 1 M NaCl and from 40 to 100% within 5 min at a flow rate of 1 mL/min. Fractions with UPO activity *versus* DMP were harvested, concentrated, and dialyzed against buffer Tris HCl 20 mM at pH 7.8 (buffer B) and loaded onto a strong cation-exchange column (HiTrap QFF GE Healthcare) pre-equilibrated with buffer B. The proteins were eluted with a linear gradient from 0 to 20% (in 40 min) of buffer B with 1 M NaCl and from 20 to 100% within 5 min at a flow rate of 1 mL/min. The fractions with UPO activity *versus* DMP were pooled, dialyzed against buffer potassium phosphate 10 mM at pH 7.0, concentrated, and stored at 4 °C.

### Biochemical Characterization

The pH activity profile of FuncLib mutants with different substrates was calculated with appropriate dilutions of enzyme samples, prepared in such a way that aliquots of 20 μL gave rise to a linear response in kinetic mode. The optimum pH activity was determined using 100 mM Britton and Robinson buffer at different pH values (from 2.0 to 9.0) with H_2_O_2_ (2 mM), DMP (1 mM), ABTS (1 mM), or NBD (1 mM, 15% acetonitrile v/v). The activities were measured in triplicate, and the relative activity (in %) is based on the maximum activity at a certain pH for each enzyme.

pH stability profiles of FuncLib mutants were calculated with appropriate pure enzyme dilutions incubated at different times over a range of pH values in 20 mM Britton and Robinson buffer (from 2.0 to 9.0). Samples were removed at different times (0, 0.5, 1.5, 3, 6, and 22 h), and activity was measured in 100 mM phosphate buffer pH 6.0 with DMP (1 mM) and H_2_O_2_ (2 mM). The experiments were performed in triplicate and measured in kinetic mode. The residual activity was related to the 100% initial activity.

Kinetic thermostability of PROSS and Funclib mutants was estimated by assessing their *T*_50_ values using 96-well gradient thermocyclers (Mycycler, Bio-Rad, USA). *T*_50_ was defined as the temperature at which the enzyme maintained 50% of its initial activity after a 10 min incubation. Appropriate UPO dilutions were prepared in such a way that 20 μL aliquots gave rise to a linear response in the kinetic mode. Then, 50 μL was used for each point in the gradient scale, and a temperature gradient profile ranging from 30 to 70 °C was established as follows (in °C): 30.0, 31.6, 34.6, 39.5, 45.0, 46.8, 49.8, 54.4, 59.9, 64.8, 68.0, and 70.0. After a 10 min incubation, samples were chilled out on ice for 10 min and further incubated at room temperature for 5 min. Afterward, 20 μL of samples were assayed in sodium phosphate (pH 6.0, 100 mM), which contained H_2_O_2_ (2 mM) and DMP (1 mM). Reactions were performed in triplicate, and substrate oxidations were followed through spectrophotometric changes. The thermostability values were deduced from the ratio between the residual activities incubated at different temperature points and the initial activity at room temperature.

Steady-state kinetic constants: ethylbenzene kinetic constants for PaDa-I, d2, d4, and d28 were estimated in 100 mM phosphate buffer pH 7.0, containing 2 mM H_2_O_2_. Reactions were performed in triplicate, and substrate oxidations were followed through spectrophotometric changes (ε_248_ phenylethanol = 147.3 M^–1^ cm^–1^). To calculate the *K*_m_ and *k*_cat_ values, the average *V*_max_ was represented against the substrate concentration and fitted to a single rectangular hyperbola function with SigmaPlot 10.0, in which parameter a was equal to *k*_cat_ and parameter *b* was equal to *K*_m_.

### Selectivity Reactions and Analysis (GC-FID and GC–MS)

Reactions contained 60–120 μL of supernatant from each enzyme (flask production in *S. cerevisiae* concentrated fivefold) and 2 mM of H_2_O_2_ in 100 mM potassium phosphate buffer pH 6.0 and 10 mM of substrate (**1a–5a**) in a final volume of 0.3 mL. Reactions were incubated at 30 °C shaking at 800 rpm in a Thermomixer C (Eppendorf, Germany) for 3 h.

TTNs of d2, d4, d28, and PaDa-I: 50 nM of each UPO (concentration measured using the CO difference spectrum as described elsewhere (Gomez de Santos *et al.*, 2020)) were assayed with 2 mM of H_2_O_2_ in 100 mM potassium phosphate buffer pH 6.0 and 10 mM of substrate (**3a** or **5a**). Reactions were incubated at 30 °C, shaking at 800 rpm, in a Thermomixer C (Eppendorf, Germany) for 3 h.

Reaction samples were mixed with an equal quantity of ethyl acetate and centrifuged at 11,000 rpm for 2 min. The organic phase was then pipetted out carefully, dried over Mg_2_SO_4_, and analyzed by GC–MS with a GC-2010 Plus coupled to the mass detector GC–MS-QP2020ISQ or by GC-FID (both from Shimazdu, Japan). Methods can be found in Table S5. TTNs were calculated based on calibration curves obtained from authentic standards. All reactions were performed in duplicate.

## Supporting Information Available

FuncLib strategy, PROSS mutants’ biochemical characterization, the activity profiles of FuncLibs, the heatmap of enantioselectivities, the biochemical characterization of FuncLibs in terms of pH activity and stability profiles, thermostability, spectroscospic features, PROSS mutations, the tolerate sequence space for FuncLib diversification, the ee of FuncLibs, the enantioselectivities (in %) of FuncLibs, and the GC analytical conditions (PDF)
