# Supplementary material for: Repertoire of Computationally Designed Peroxygenases for Enantiodivergent C–H Oxyfunctionalization Reactions
Source: J Am Chem Soc. 2023 Jan 23;145(6):3443–53. doi: 10.1021/jacs.2c11118 (PMC9936548; doi:10.1021/jacs.2c11118)
Supplement: Supplementary file 1 — ja2c11118_si_001.pdf [file ja2c11118_si_001.pdf]

Supplementary information for:

A repertoire of computationally designed peroxygenases for enantiodivergent C-H oxyfuntionalisation reactions

Patricia Gomez de Santos[1,2], Ivan Mateljak[2], Manh Dat Hoang[1,3], Sarel J. Fleishman[4], Frank Hollmann[5] and Miguel Alcalde[1*]

[1] Department of Biocatalysis, Institute of Catalysis, ICP-CSIC, C/ Marie Curie 2, 28049 Madrid, Spain.

[2] EvoEnzyme S.L., Parque Científico de Madrid, C/ Faraday 7, 28049 Madrid, Spain.

[3] Chair of Biochemical Engineering, Technical University of Munich, Boltzmannstr. 15, 85748 Garching, Germany.

[4] Department of Biomolecular Sciences, Weizmann Institute of Science, 7610001 Rehovot, Israel.

[5] Department of Biotechnology, Delft University of Technology, van der Massweg 9, 2629HZ Delft, The Netherlands.

* Correspondence should be addressed to: M.A. (malcalde@icp.csic.es).

Figure S1

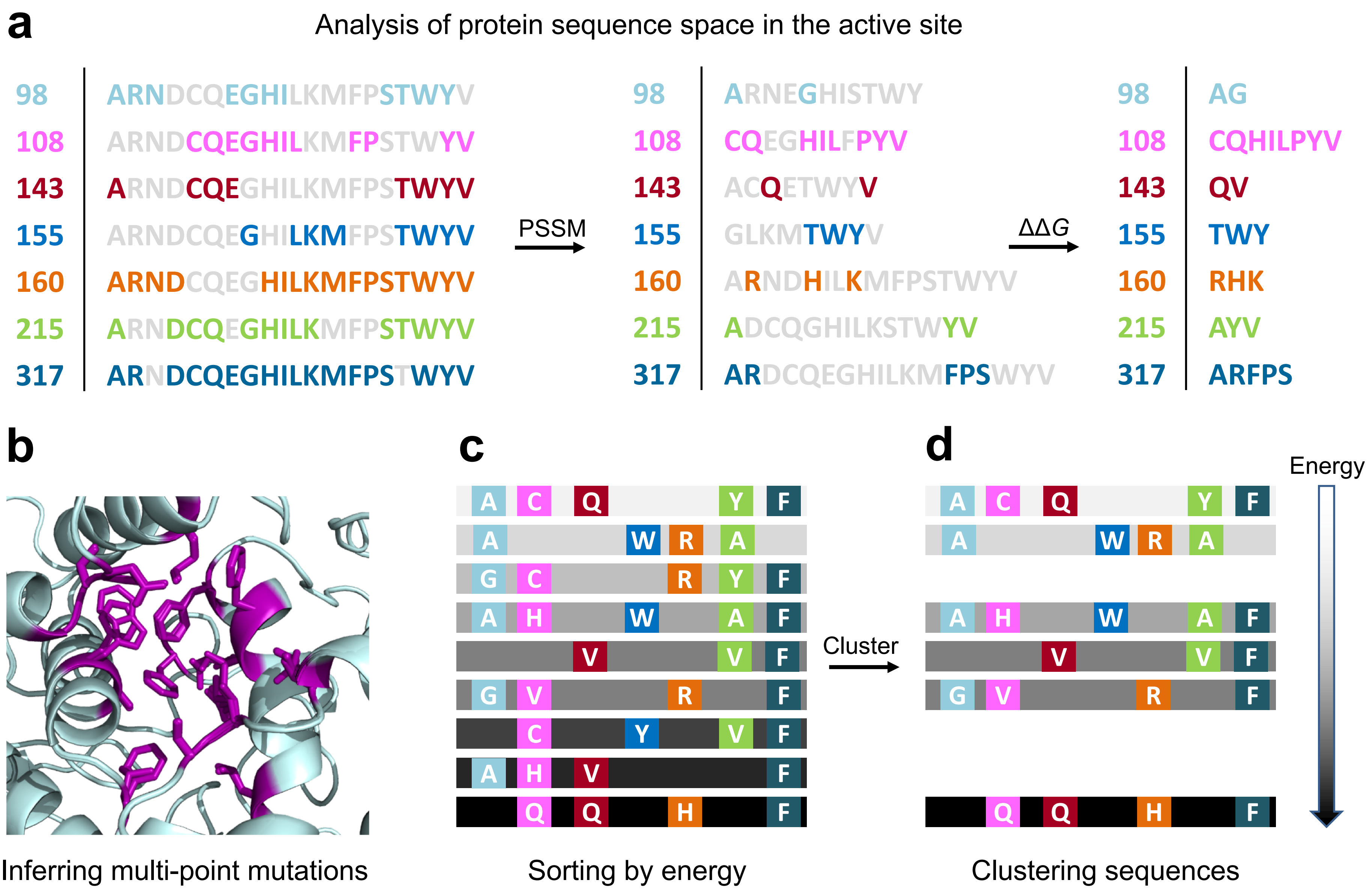

Figure S1. General workflow for FuncLib computational mutagenesis. (**a**) analysis of the sequence protein space focused on the catalytic pocket by position specific scoring matrix -PSSM- and selection of residues according to their energy in the context of the protein; (**b**) the multipoint mutations are inferred by Rosetta atomistic calculation,(**c**) sorted by energy and (**d**) clustered in specific sequences. Figure adapted from Khersonksky et al., 2018.

Figure S2

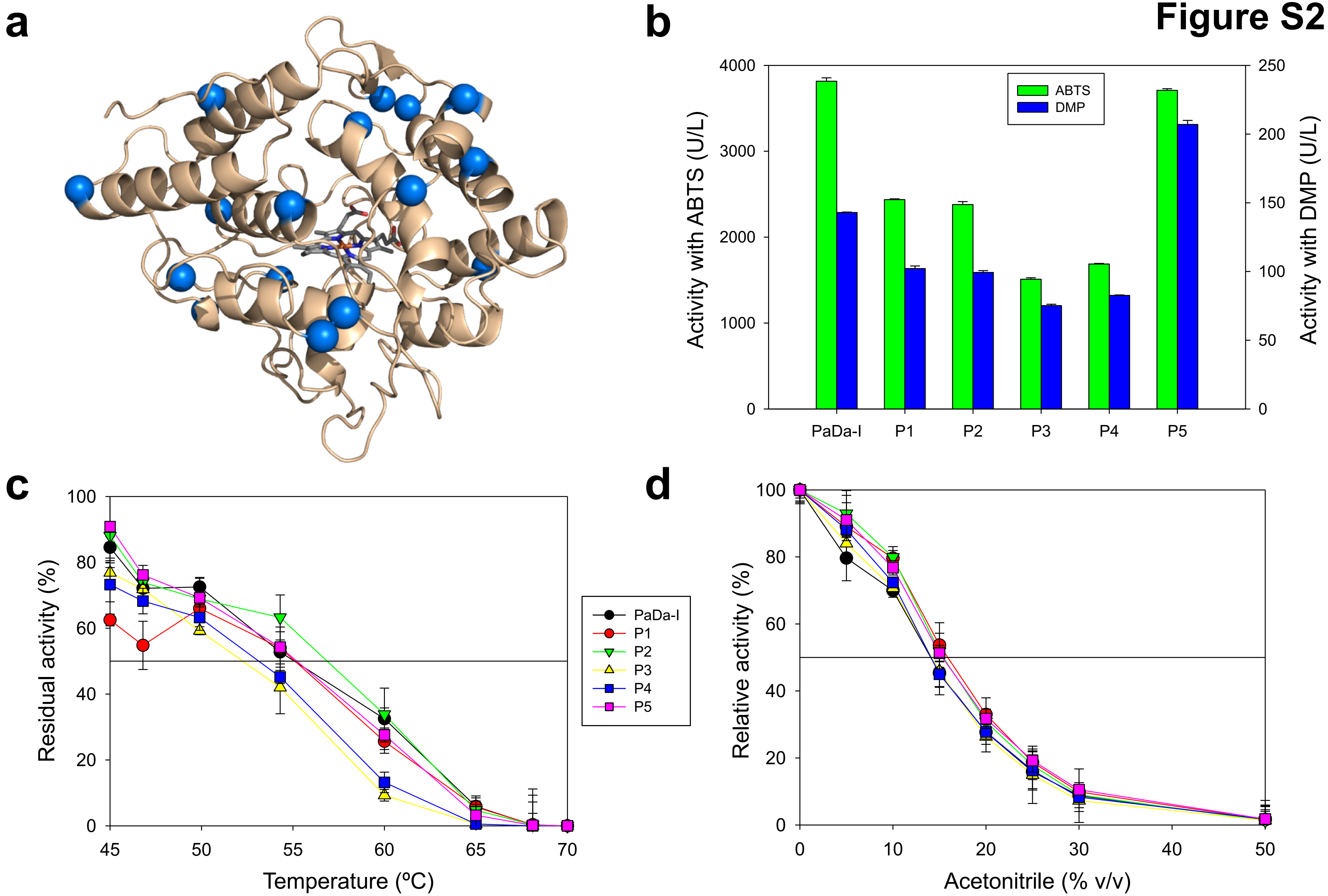

Figure S2. PROSS mutants and their biochemical properties (**a**) PROSS mutations within the UPO structure highlighted as blue spheres (figure visualized with PyMOL). (**b**) Total activity values measured with ABTS and DMP. (**c**) Thermostability (T_{50}). (**d**) Activity in increasing concentrations of acetonitrile. The activity was measured with 2 mM H_2O_2 and 1 mM ABTS in 100mM sodium phosphate/citrate buffer (pH 4.0) containing the corresponding concentration acetonitrile. Each point represents the mean and standard deviation of 3 independent experiments.

Figure S3

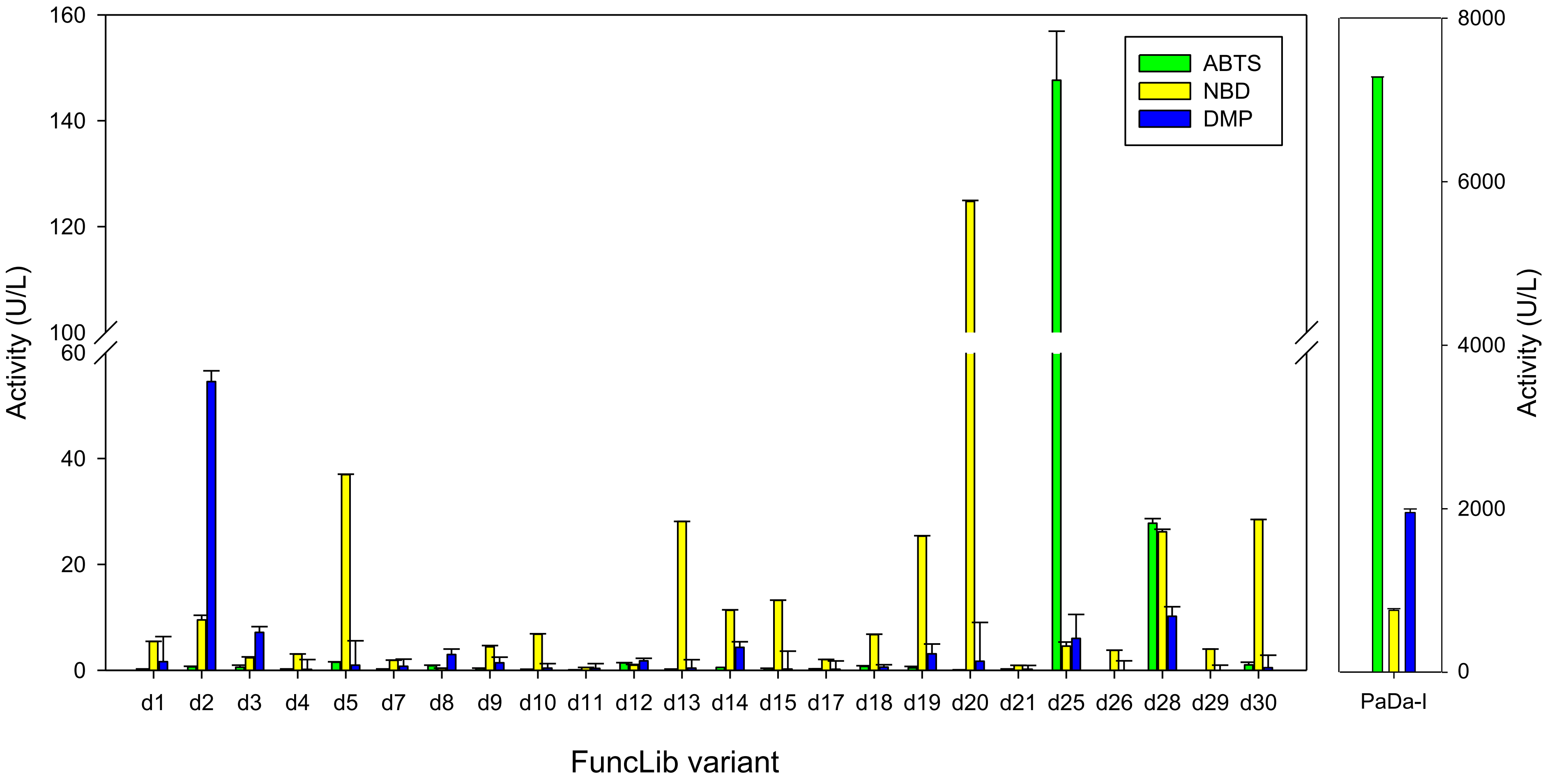

Figure S3. Activity profile of FuncLib variants and PaDa-I functional expressed by *S. cerevisiae.* Activities were measured with DMP, ABTS (peroxidase substrates) and NBD (peroxygenase substrate).

Figure S4

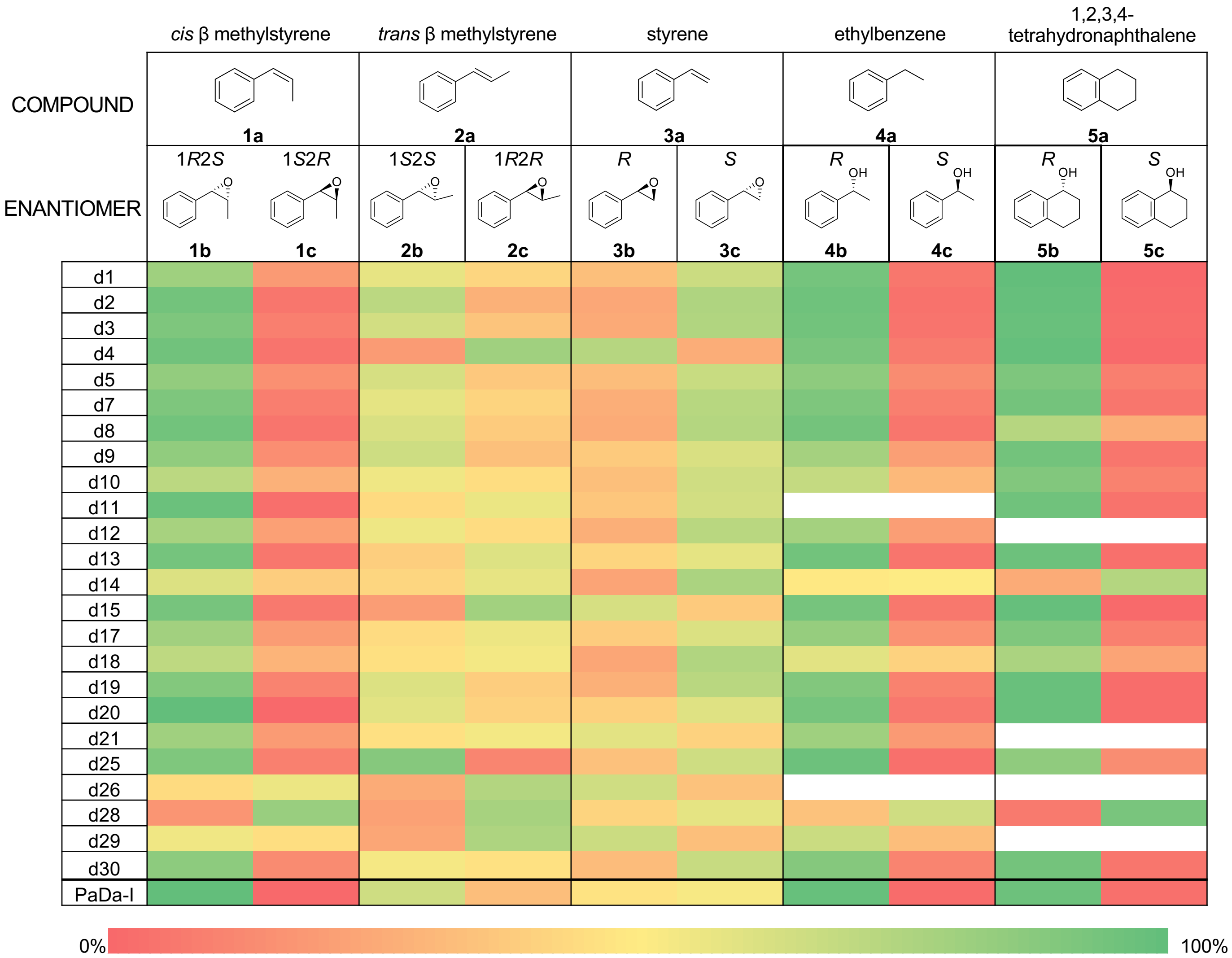

Figure S4. Heat map of different enantioselectivities of FuncLib designs compared to parental type PaDa-I. See also **Supplementary Tables S3, S4**.

Figure S5

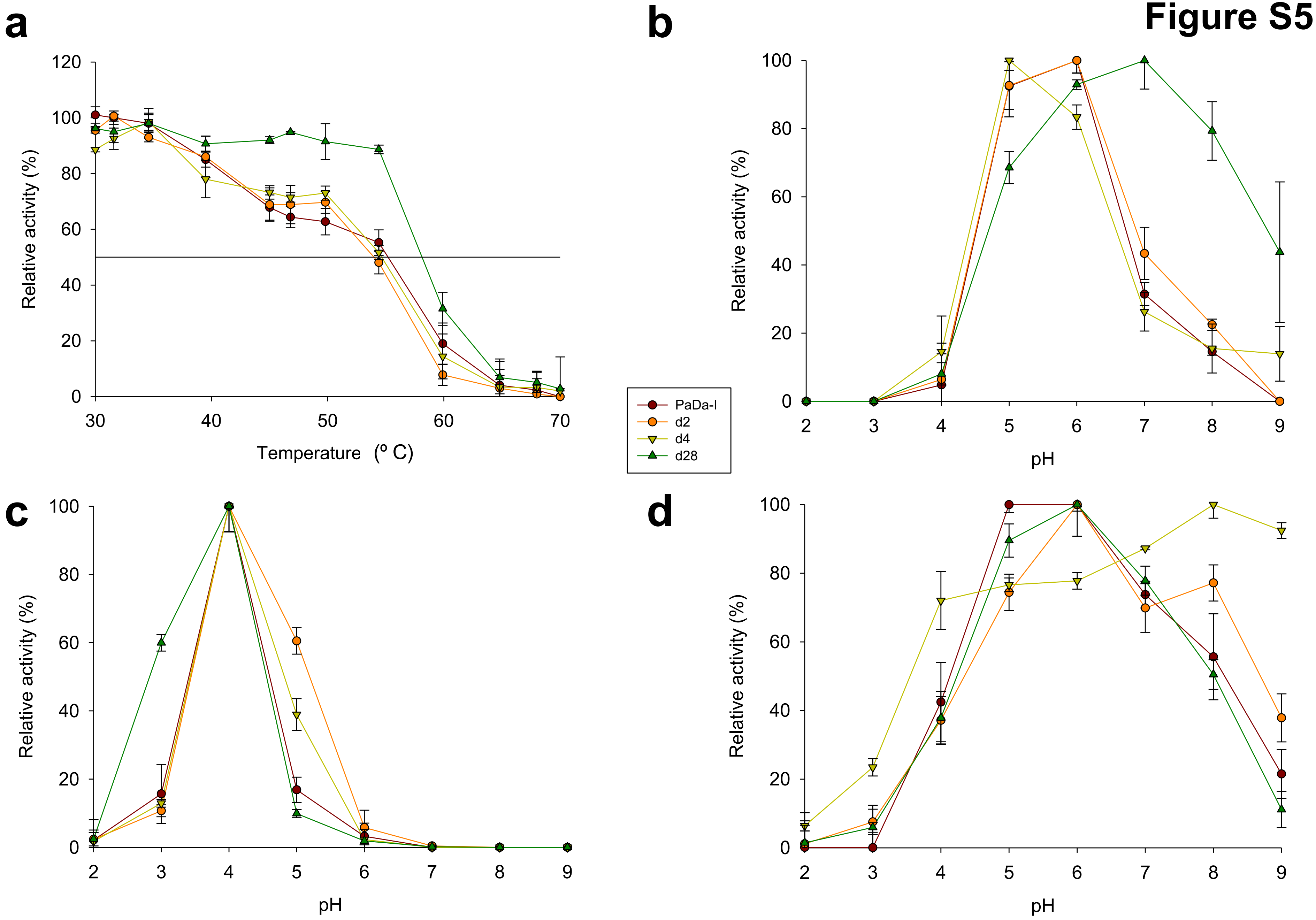

Figure S5. Biochemical characterization of FuncLib variants and PaDa-I. (**a**) Kinetic thermostability (T_{50}) and pH activity profiles measured with 1 mM NBD (**b**), 1 mM ABTS (**c**) and 1 mM DMP (**d**) in 100mM Britton-Robinson buffer at different pHs from 2.0 to 9.0 containing 2 mM H_2O_2. Each point represents the mean and standard deviation from 3 independent experiments.

Figure S6

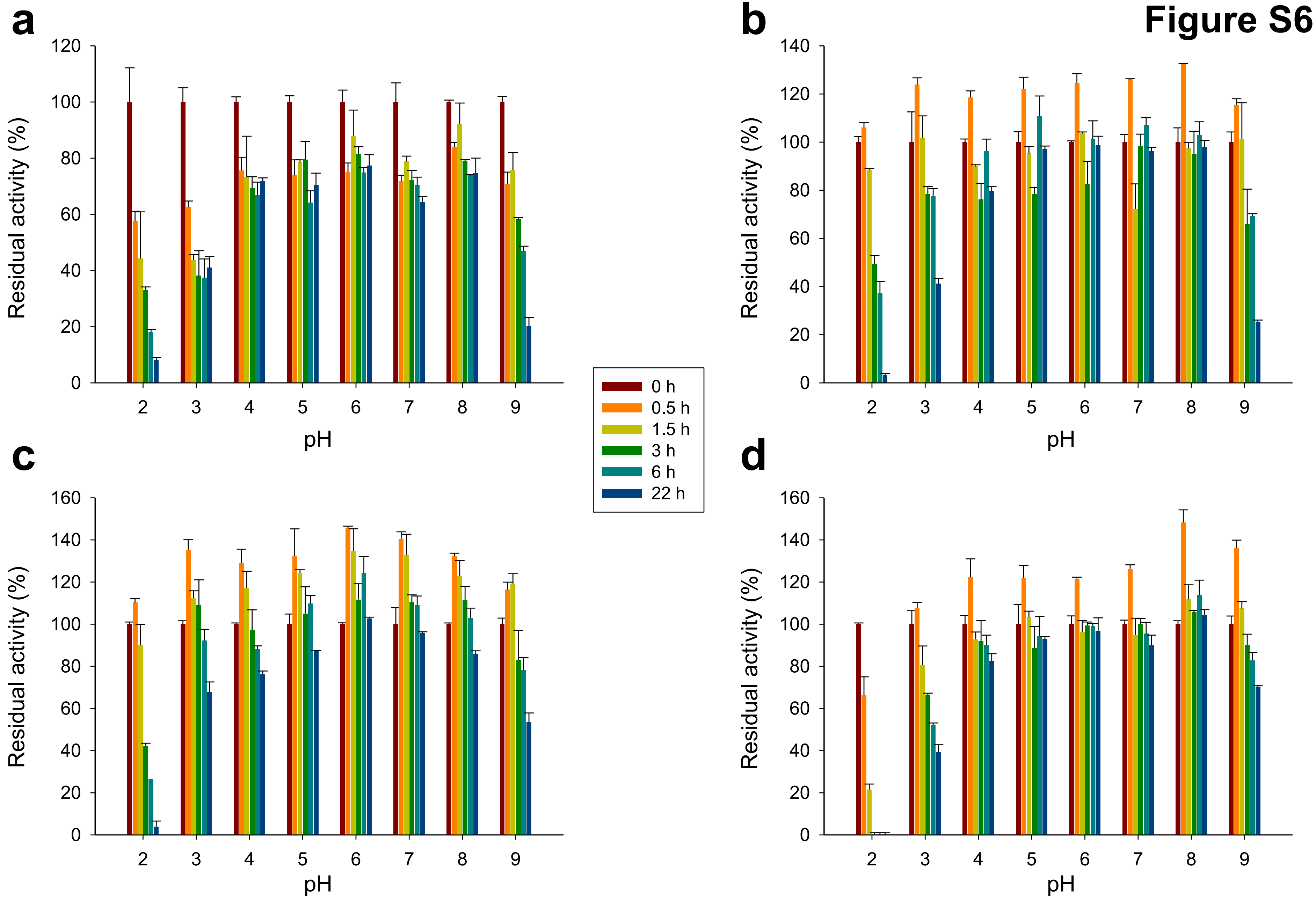

Figure S6. Biochemical characterization. pH stability of FuncLib variants and PaDa-I. (**a**) PaDa-I, (**b**) d2 mutant, (**c**) d4 mutant, (**d**) d8 mutant. Appropriate enzyme dilutions were incubated at different times over a range of pH values in 20mM Britton-Robinson buffer. Aliquots were removed at different times to measure activity with 5mM veratryl alcohol and 2mM H_2O_2 in 100mM phosphate buffer (pH 7.0) at room temperature. Each point represents the mean and standard deviation from 3 independent experiments.

Figure S7

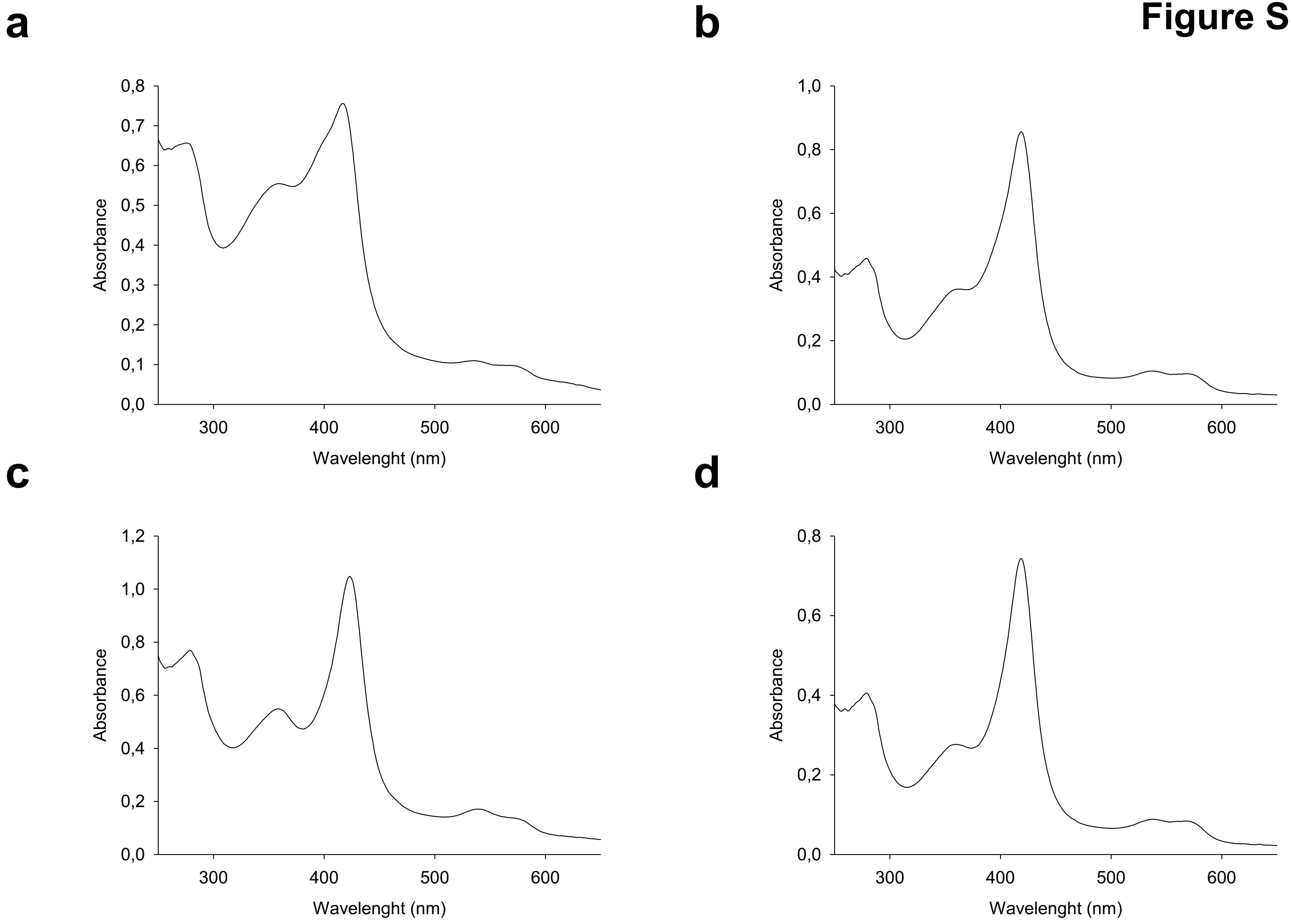

Figure S7. Spectroscopic characteristics of (**a**) PaDa-I, (**b**) d2, (**c**) d4 and (**d**) d28.

Figure S8

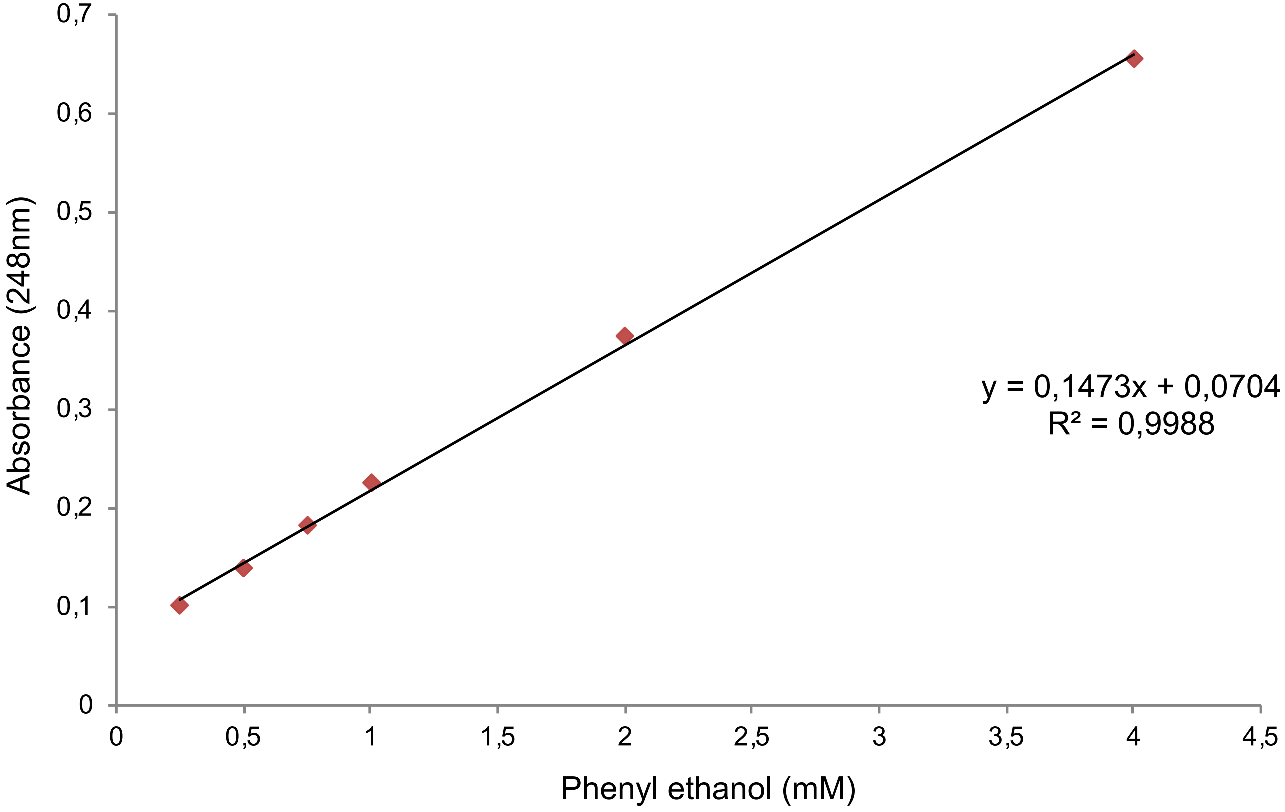

Figure S8. Experimental determination of the molar extinction coefficient for phenylethanol.

Table S1. Pross mutants selected in this study.

PROSS mutant	Mutations
P1	N11D, Q72D, G166A, V298G
P2	N11D, Q72D, G166A, K261G, V298G
P3	N11D, Q72D, R97G, S111T, G166A, F190Y, T242D, Q249N, K261G, N286L, V298G
P4	N11D, Q72D, R97G, S111T, E146Q, G166A, F190Y, T242D, Q249N, K261G, I262V, N286L, V298G
P5	P5: N11D, S12N, Q72D, D91N, R97G, R100S, S111T, E146Q, G166A, F190Y, T242D, Q249A, K261G, I262V

Table S2. Positions selected for FuncLib diversification and their tolerated sequence space.

Positions	Sequence space
69	FL
76	FWY
77	AI
121	FIWY
188	F
191	F
192	TACEHILMQSV
199	FALY
240	ST
241	GAILMNSTVY
244	VAIT
280	MAT
314	GA
315	VY
316	ADEHIKNPQSTV

Table S3. Enantiomeric excess (*ee*) of FuncLib designs and parental type PaDa-I.

	cis β methylstyrene oxide	*trans* β methylstyrene oxide	Styrene oxide	1-phenyl ethanol			α-tetralol	
							1-ol	2-ol
	(**1b**) *ee* (1*R*2*S*) [%]	(**2b**) *ee* (1*S*2*S*) [%]	(**3b**) *ee* (*R*) [%]	(**4b**) *ee* (*R*) [%]	*ortho*	*para*	(**5b**) *ee* (*R*) [%]	
d1	62	16	-34	88		16	100	7
d2	90	44	-52	92		5	98	2
d3	82	30	-50	90		11	96	2
d4	92	-62	48	86		2	98	
d5	70	26	-36	72		9	82	7
d7	84	18	-46	82	63		90	
d8	90	24	-48	90	39		46	
d9	70	32	-24	58			90	8
d10	44	10	-34	38		48	80	14
d11	96	-12	-28	n.d.			92	
d12	56	12	-46	58			n.d.	
d13	88	-22	-16	90	6		94	2
d14	22	-16	-54	-2		18	-48	11
d15	88	-60	26	88			98	
d17	60	-12	-24	68			82	5
d18	42	-8	-52	20		25	54	13
d19	80	22	-46	80			96	4
d20	100	18	-20	88			96	1
d21	60	-8	18	62			n.d.	
d25	82	78	-32	94			72	
d26	-12	-50	32	n.d.			n.d.	
d28	-66	-59	-18	-32			-86	8
d29	10	-52	34	34			n.d.	
d30	74	8	-36	78		6	90	6
PaDa-I	100	32	-6	98			94	

Table S4. Enantioselectivities of FuncLib designs and parental type PaDa-I. [a]% of total product. *% of each enantiomer from the remaining product formed.

	cis β methylstyrene (**1a**)		*trans* β methylstyrene (**2a**)		Styrene (**3a**)		Ethylbenzene (**4a**)				1,2,3,4 tetrahydronaphthalene (**5a**)		
											1-ol		2-ol[a]
	(**1b**) 1*R*2*S*	(**1c**) 1*S*2*R*	(**2b**) 1*S*2*S*	(**2c**) 1*R*2*R*	(**3b**) *R*	(**3c**) *S*	(**4b**) *R**	(**4c**) *S**	*ortho*[a] -OH	*para*[a] -OH	(**5b**) *R**	(**5c**) *S**	
d1	81	19	58	42	33	67	94	6		16	100	0	7
d2	95	5	72	28	24	76	96	4		5	99	1	2
d3	91	9	65	35	25	75	95	5		11	98	2	2
d4	96	4	19	81	74	26	93	7		2	99	1	
d5	85	15	63	37	32	68	86	14		9	91	9	7
d7	92	8	59	41	27	73	91	9	63		95	5	
d8	95	5	62	38	26	74	95	5	39		73	27	
d9	85	15	66	34	38	62	79	21			95	5	8
d10	72	28	55	45	33	67	69	31		48	90	10	14
d11	98	2	44	56	36	64	---	---			96	4	
d12	78	22	56	44	27	73	79	21			---	---	
d13	94	6	39	61	42	58	95	5	6		97	3	2
d14	61	39	42	58	23	77	49	51		18	26	74	11
d15	94	6	20	80	63	37	94	6			99	1	
d17	80	20	44	56	38	62	84	16			91	9	5
d18	71	29	46	54	24	76	60	40		25	77	23	13
d19	90	10	61	39	27	73	90	10			98	2	4
d20	100		59	41	40	60	94	6			98	2	1
d21	80	20	46	54	59	41	81	19					
d25	91	9	89	11	34	66	97	3			86	14	
d26	44	56	25	75	66	34	---	---			---	---	
d28	17	83	22	78	41	59	34	66			7	93	8
d29	55	45	24	76	67	33	67	33			---	---	
d30	87	13	54	46	32	68	89	11		6	95	5	6
PaDa-I	100	0	66	34	47	53	99	1			97	3	

Table S5. GC analytics.

Compound	Column temperature	Program/ gradient retention	Retention time
**1a/1b/1c**	Lipodex E (Macherey-Nagel)<br>(50 m × 0.25 mm × 0.25 µm)<br>carrier gas: He	120°C hold 5.0 min<br>10°C/min to 210°C hold 1.0 min	8.67 min *cis*-β-methylstyrene<br>11.18 min (1*R*,2*S*)-*cis*-β-methylstyrene oxide<br>11.60 min (1*S*,2*R*)-*cis*-β-methylstyrene oxide
**2a/2b/2c**	Lipodex E (Macherey-Nagel)<br>(50 m × 0.25 mm × 0.25 µm)<br>carrier gas: He	120°C hold 3.0 min<br>2°C/min to 150°C hold 1.0 min<br>20°C/min to 180°C	9.80 min *trans*-β-methylstyrene<br>12.97 min (1*S*,2*S*)-*trans*-β-methylstyrene oxide<br>13.25 min (1*R*,2*R*)-*trans*-β-methylstyrene oxide
**3a/3b/3c**	CP-Chirasil-Dex-CB (Agilent)<br>(25 m × 0.32 mm × 0.25 µm)<br>carrier gas: He	100°C hold 8.0 min,<br>20°C/min to 220°C hold 1.0 min	2.15 min styrene<br>6.05 min (*R*)-styrene oxide<br>6.50 min (*S*)-styrene oxide
**4a/4b/4c**	CP-Chirasil-Dex-CB (Agilent)<br>(25 m × 0.32 mm × 0.25 µm)<br>carrier gas: He	120°C hold 2.6 min,<br>15°C/min to 135°C hold 3.3 min<br>25°C/min to 225°C hold 1.0 min	1.49 min ethylbenzene<br>4.70 min (*R*)-1-phenylethanol<br>4.91 min (*S*)-1-phenylethanol<br>6.82 min 2-ethylphenol<br>7.36 min 4-ethylphenol
**5a/5b/5c**	CP-Chirasil-Dex-CB (Agilent)<br>(25 m × 0.32 mm × 0.25 µm)<br>carrier gas: He	120°C hold 3.0 min,<br>5°C/min to 140°C hold 12.0 min<br>25°C/min to 190°C hold 2.0 min	4.3 min 1,2,3,4-tetrahydronaphthalene<br>13.56 min (*S*)-α-tetralol<br>13.92 min (*R*)-α-tetralol<br>15.77 min 1,2,3,4-tetrahydro-2-naphthol
